# Leveraging learned monocular depth prediction for pose estimation and mapping on unmanned underwater vehicles

**DOI:** 10.3389/frobt.2025.1609765

**Published:** 2025-06-26

**Authors:** Marco Job, David Botta, Victor Reijgwart, Luca Ebner, Andrej Studer, Roland Siegwart, Eleni Kelasidi

**Affiliations:** ^1^ Autonomous Systems Lab, Institute of Robotics and Intelligent Systems, Department of Mechanical and Process Engineering, ETH, Zurich, Zurich, Switzerland; ^2^ Department of Mechanical and Industrial Engineering, Norwegian University of Science and Technology (NTNU), Trondheim, Norway; ^3^ Tethys Robotics, Zurich, Switzerland; ^4^ Aquaculture Robotics and Automation Group, SINTEF Ocean, Trondheim, Norway

**Keywords:** localization, mapping, UUVs, depth prediction, aquaculture

## Abstract

This paper presents a general framework that integrates visual and acoustic sensor data to enhance localization and mapping in complex, highly dynamic underwater environments, with a particular focus on fish farming. The pipeline enables net-relative pose estimation for Unmanned Underwater Vehicles (UUVs) and depth prediction within net pens solely from visual data by combining deep learning-based monocular depth prediction with sparse depth priors derived from a classical Fast Fourier Transform (FFT)-based method. We further introduce a method to estimate a UUV’s global pose by fusing these net-relative estimates with acoustic measurements, and demonstrate how the predicted depth images can be integrated into the wavemap mapping framework to generate detailed 3D maps in real-time. Extensive evaluations on datasets collected in industrial-scale fish farms confirm that the presented framework can be used to accurately estimate a UUV’s net-relative and global position in real-time, and provide 3D maps suitable for autonomous navigation and inspection.

## 1 Introduction

The aquaculture industry has seen rapid growth over the last decades. Fish farming, in particular, has emerged as a vital source of the global seafood supply (FAO, 2018). This growth, however, presents new challenges in terms of ensuring efficient, safe, and sustainable operations (Føre et al., 2018). Fish farming often involves a significant amount of manual labor, which can be physically demanding and dangerous. Tasks such as net inspection, maintenance, and repairs expose workers to hazardous underwater conditions, including rough seas, low visibility, and the presence of potentially harmful marine life. Addressing some of these problems, the interest in robotic systems for aquaculture has also grown significantly in recent years (Kelasidi and Svendsen, 2023).

Current robotic solutions often involve the use of manually operated UUVs, such as Remotely Operated Vehicles (ROVs) for inspection and intervention activities in fish farms, which are expensive to deploy as they can only be operated by highly trained ROV pilots (Føre et al., 2018; Kelasidi and Svendsen, 2023). As the number of fish farms increases, and with the trend toward deploying these farms in increasingly remote locations (Bjelland et al., 2015), the automation of such tasks becomes crucial for enhancing operational efficiency (Schellewald et al., 2021). Autonomous UUVs offer a promising solution to these challenges, reducing weather and manual labor-dependent risks and allowing for more efficient and sustainable procedures (Kelasidi and Svendsen, 2023). However, deploying autonomous UUVs in fish farms requires robust methods for localization and mapping in net pens, which remains an open research problem.

Traditional UUV navigation systems rely heavily on acoustic sensors, such as echo-sounders, Ultra-short baseline (USBL) acoustic positioning systems, and Doppler Velocity Loggers (DVLs) (Fossen, 2011; Kelasidi and Svendsen, 2023). While effective in many underwater scenarios, these sensors face significant limitations in fish farms. The permeable nature of fish nets can lead to weak or distorted acoustic reflections, resulting in poor target signal strengths (Amundsen et al., 2022). The high density of fish within these environments further disturbs acoustic measurements (Rundtop and Frank, 2016). Recent research has explored the use of stereo vision systems and image processing techniques to enhance UUV localization (Skaldebø et al., 2023). Stereo cameras, for example, have been employed to achieve 3D spatial awareness, which is crucial in environments where precise positioning relative to net structures is required. Techniques such as the FFT-based method for relative pose estimation in net pens (Schellewald et al., 2021) and the TRU-depth network for depth estimation (Ebner et al., 2024) have shown promising results in underwater applications. Additionally, methods for pose estimation using laser triangulation have demonstrated accuracy comparable to DVL systems at a fraction of the cost, making them a viable option for short-distance ranging in fish farming environments. However, these methods also encounter similar issues with interference from fish and are additionally sensitive to light changes (Bjerkeng et al., 2023).

Simultaneous Localization and Mapping (SLAM) is widely used for mobile and aerial robotics applications (Ebadi et al., 2024). Map representations generally fall into two categories: sparse feature-based methods and dense methods. Sparse representations, which rely on a small set of distinctive features, are well-suited for odometry and localization tasks and efficiently scale to large environments (Abaspur Kazerouni et al., 2022). In contrast, dense methods–including point clouds, surfels, meshes, and volumetric maps–provide rich representations of the environment’s geometry, making them ideal for navigation and inspection tasks (Mascaro and Chli, 2024). Volumetric maps (Izadi et al., 2011; Oleynikova et al., 2017; Vespa et al., 2018; Duberg and Jensfelt, 2020; Reijgwart et al., 2023) facilitate safe navigation in unknown environments, as they can represent objects of arbitrary shape and explicitly differentiate observed free space from unobserved space. In this work, we adopt wavemap (Reijgwart et al., 2023) due to its hierarchical structure and advantageous mathematical properties, enabling state of the art accuracy and efficiency in mapping and downstream applications, such as reactive collision avoidance (Reijgwart et al., 2024).

Compared to land and flying robots, the majority of mapping approaches in the underwater domain rely on acoustic sensors (e.g., imaging sonars) to generate the map of the inspected areas (Xu et al., 2022; Jiang et al., 2019; Arnesen et al., 2018). Lately, interest has increased in integrating visual, inertial, and acoustic sensors for underwater SLAM (Kelasidi and Svendsen, 2022). SVin2 is a state-of-the-art underwater SLAM method fusing camera, sonar, IMU and a barometer (Wang et al., 2023). However, it struggles to provide accurate results in low-texture environments. Joshi and Rekleitis (2024) proposed a visual-inertial odometry method fusing measurements from the magnetometer. Song et al. (2024) proposed TURTLMap, and investigated a real-time localization and dense mapping for UUVs for low texture applications by also integrating DVL measurements, yet it was only tested in a controlled lab environment. Cardaillac et al. (2024) presented an offline approach utilizing multi-beam sonar data for mapping of net pens. However, very limited research exists on SLAM for autonomous operations in dynamic underwater environments such as fish farms, which remains an open research challenge.

To address these challenges, this paper investigates monocular vision-based methods to: 1) obtain point-wise distance and orientation estimates with respect to a flexible, deformable net structure, 2) predict dense depth images from monocular visual data for subsequent use in pose estimation, UUV navigation, and 3D mapping in dynamic environments, 3) estimate the global pose of an UUV within the net pen by fusing the relative pose observations with the robot’s additional onboard sensors, and 4) create detailed 3D maps of the net-pen environments for navigation and to identify irregularities such as holes, or biofouling in industrial-scale fish farms. Evaluations on large datasets recorded in industrial scale fish farms showcase the potential of combining learning-based depth prediction methods (Ebner et al., 2024; Job et al., 2024), FFT based net-relative pose estimation method (Schellewald et al., 2021), and wavemap (Reijgwart et al., 2023) to estimate both net-relative and global position of the UUV, and produce accurate maps, even in such dynamic environments.

## 2 Methods and theoretical background

This section provides a brief overview of the proposed underwater localization and mapping framework, which allows UUVs to operate in dynamic underwater environments.

### 2.1 Fast Fourier Transform (FFT)-based method

Schellewald et al. (2021) proposed an FFT-based method to estimate the pose of a net structure relative to the camera attached on an UUV, utilizing only monocular visual information. In particular, this method analyzes the frequency spectrum of captured images to determine the distance and orientation of the camera based on characteristic regular patterns within the image, as well as the knowledge of the actual dimensions of the net squares. In order to analyze the image I in the frequency domain, I is converted using the Fourier Transform (FT), denoted as F(I) . To efficiently compute F(I) , the FFT-based method utilizes the FFT algorithm, developed by Cooley and Tukey (1965), which computes the Discrete Fourier Transform (DFT) of an N×N image (u,v∈[0,N−1]) using the following expression:

$$F\left(u,v\right)=\frac{1}{N}\overset{N-1}{\underset{x=0}{\sum }}\overset{N-1}{\underset{y=0}{\sum }}I\left(x,y\right)e^{-i2\pi \left(\frac{ux}{N}+\frac{vy}{N}\right)},$$ (1)

where $N=2^{m}$ and m being an integer.

**FIGURE 1:**
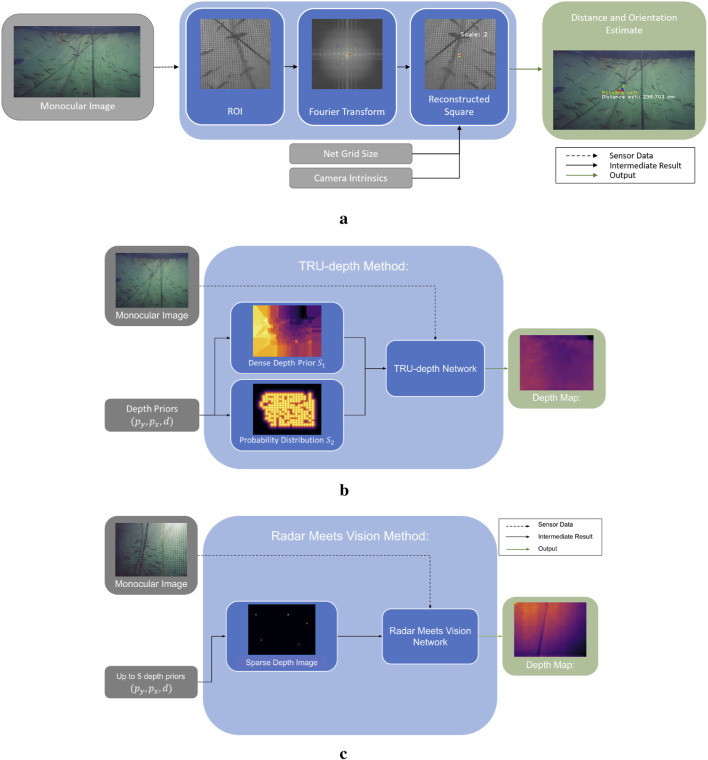
Overview of the Dense Image Prediction Methods. The TRU-depth method (Ebner et al., 2024) uses the available depth priors and converts them to two dense network inputs. The output is a dense metric depth map. The Radar Meets Vision method (Job et al., 2024) limits the number of input depth prior points to five. Using a dense representation of the sparse depth priors and the monocular image, the network predicts dense metric depth as well. **(a)** Overview of the FFT-based method for net-relative pose estimation. **(b)** Overview of the TRU-depth method. **(c)** Overview of the Radar Meets Vision method.

As proven by Schellewald et al. (2021), repeated textures, such as a net’s squares, lead to peaks within the FT’s magnitude image |F{I}| (see Figure 1a). This method therefore splits the image into Regions of Interest (ROIs), computes each region’s FFT, and counts how many of its local maxima lie on a regular grid. If a certain threshold is exceeded, the ROI is assumed to contain a subsection of the net. The grid of local maxima can then be used to reconstruct the base vectors of a single parallelogram that approximates the mesh squares. In combination with knowledge of the net’s grid size and the camera’s intrinsics, this parallelogram can, in turn, be used to estimate the camera’s net-relative translation and orientation. Note that the original paper (Schellewald et al., 2021) concludes that we do not need to define the threshold for each region since the FFT-based method does not depend on a fixed threshold value, as it detects regular grid patterns based on local maxima in the frequency domain. Therefore, the method performs well as long as the regular structure is (partly) visible in the region, even if the contrast in the areas is low. In addition, we need to emphasize that while generic nets are flexible in principle, commercial salmon pens are typically more rigid and tend to maintain (local) regularity, especially over small ROIs used by the suggested method. For further details, see Schellewald et al. (2021).

### 2.2 Depth image prediction methods

Several depth image prediction methods have been proposed in the scientific community suited for applications in the underwater domain, such as DepthAnything (Yang et al., 2024a), RAFT-Stereo (Lipson et al., 2021), UDepth (Yu et al., 2023), TRU-depth (Ebner et al., 2024) and Radar Meets Vision (RMV) (Job et al., 2024). This paper adapts the TRU-depth and RMV methods to obtain depth image predictions of the inspected net pen, as both methods enable metrically scaled real-time depth estimation and thus surpass the inherent problem related to scale ambiguity of monocular camera systems, while also addressing the issue of generalizability of supervised deep learning models to predict metric depth from monocular RGB images.

#### 2.2.1 TRU-depth method

TRU-depth is a deep learning-based approach that generates dense depth images from monocular RGB images by fusing additional sparse depth information, such as depth priors from external measurements. The network utilizes these sparse depth priors to mitigate scale ambiguity, producing metrically scaled depth images. Figure 1b illustrates the steps of the TRU-depth method. In the first step, the sparse depth measurements are converted into a dense format that can be used as a network input. To achieve this, the method uses two types of dense prior maps. The first map, denoted as $S_{1}$ , is created through nearest-neighbor interpolation, where each pixel in the map is assigned the depth of its closest key point. The second map, $S_{2}$ , represents the probability distribution of distances from each pixel to the nearest key point, modeled as a normal distribution (see Figure 1b). These maps are concatenated into a dual-channel image and fed into the depth prediction network together with the monocular image.

The network architecture combines a lightweight encoder-decoder backbone with a vision transformer. The encoder-decoder, based on MobileNetV2, processes the input image and the dense prior maps, extracting features at various resolutions. The vision transformer then refines these features by dividing the depth range into adaptive bins and predicting the depth values based on the bin probabilities. This approach allows for efficient and accurate depth estimation.

The training of the network involves several loss functions to ensure robust performance: a) A Root Mean Squared Error loss function to guide the model in learning accurate metric scale predictions, b) A Scale Invariant Logarithmic Loss to balance the focus between close-range and distant depth accuracy, and c) A Chamfer Distance Loss to regularize the bin sizes, ensuring that they align with the actual depth distribution in the images. In summary, the TRU-depth method significantly improves depth prediction accuracy by effectively integrating sparse depth priors into a lightweight real-time deep learning framework, making it suitable for deployment on mobile and embedded systems, and therefore highly relevant in context of the investigated scenario. For more detailed information, we refer to the original paper (Ebner et al., 2024).

#### 2.2.2 Radar meets vision method

Job et al. (2024) recently proposed a method for learning-based depth image prediction using sparse priors. The method is designed for millimeter-wave radar sensors, which have become increasingly used in ground and aerial robotics applications. Since the point clouds produced by such low-cost radar sensors are relatively sparse, the method is designed to operate with a low number of point priors: five or fewer points are supplied during training. Radar sensors are generally not designed for underwater usage, given the conductivity of water. However, the method described by Job et al. (2024) does not make strict assumptions specific to the radar modality and generalizes well to depth priors from other sources.

Figure 1c provides a high-level overview of this method. The input data for this method are comparable to the TRU-depth method (Ebner et al., 2024) and consist of a single, 640×480 pixel RGB image concatenated along the channel axis with the single-channel sparse depth image. As the network is tailored to a few priors; it is sufficient to sample up to five points from the available depth prior points. If there are less than or equal to five points available, all points can be used directly. If there are more than five depth prior points, a greedy furthest-point sampling algorithm is applied to maximize the distance between the priors in the image plane. The intuition behind this selection method is to maximize the depth prior coverage across the scene.

In a second stage, the selected points are projected into the image plane using the camera’s calibration and converted into a densified depth image, following the process described by Job et al. (2024). The prior points are expanded to a circular shape in the image plane. This expansion accounts for the inherent uncertainty of the actual location for the prior points. This four-channel image is input into the network, split into 14×14 pixel patches, following the architecture of Yang et al. (2024b). Multiple patches can use the scale information by extending the prior points to a 5-pixel radius circular shape. The patches are fed into an image encoder, and the encoder’s embeddings are used in a transformer-style network. The decoder produces metric depth image out of a two channel output image. We refer to the original study for more details on the implementation and considerations (Job et al., 2024).

### 2.3 Wavemap method

Mapping is crucial for UUVs operating in underwater environments, ensuring safe navigation and facilitating inspection tasks. Real-time mapping enables the robot to localize, autonomously explore the environment or plan paths to specific points of interest, and avoid collisions along the way. Additionally, detailed maps created by UUVs are valuable for inspection purposes, as they accurately document spatial relationships among underwater structures, including the net and its supporting elements. Beyond single inspections, maps can be compared across multiple missions to monitor environmental changes and assess structural conditions over time.

Volumetric maps are particularly well-suited to fish-farm operations. Unlike maps based on sparse features or geometric primitives, volumetric representations accurately capture the environment’s geometry and can model obstacles of arbitrary shape. Compared to dense point-cloud, surfel, or mesh-based maps, volumetric maps explicitly distinguish observed free space from unobserved regions. This distinction is crucial in practice, as the robot’s limited sensor field of view and occlusions caused by fish and infrastructure often render large sections of its immediate surroundings unobservable. Explicit modeling of unobserved regions allows human operators or autonomous algorithms to reason about their potential traversability or exploration value, while also accounting for possibly hidden obstacles. Thus, volumetric maps enable safe UUV deployments in fish farms without relying on accurate prior maps.

This paper uses wavemap (Reijgwart et al., 2023), a volumetric mapping framework that simultaneously achieves state-of-the-art accuracy, memory efficiency, and computational efficiency. It can fuse depth images, point clouds, and additional sparse depth measurements to generate a 3D occupancy map in real time. The method is specifically designed to handle the large amounts of data generated by modern robotic sensors, addressing this challenge by leveraging multi-resolution analysis using wavelets.

The core idea behind wavemap is to represent and update the occupancy grid in a compressed wavelet space. The coefficients obtained from Haar-wavelet decomposition align naturally with an octree structure, enabling efficient hierarchical storage. As new sensor data is received, the map’s occupancy probabilities are updated using a linear log-odds formulation. Because the wavelet transform itself is linear, updates can be performed directly in the compressed domain, avoiding costly decompression and recompression steps. Additionally, wavelet-based encoding implicitly keeps all resolution levels synchronized. This significantly improves upon prior multi-resolution frameworks, such as Octomap (Arnesen et al., 2018) or Supereight (Vespa et al., 2018), which update the occupancy at one resolution per point in space and require all remaining resolution levels to be synchronized explicitly.

Wavemap leverages its implicitly synchronized, hierarchical structure to perform measurement updates in a coarse-to-fine manner. By deriving error bounds through multi-resolution analysis, wavemap dynamically determines the necessary resolution at each point, focusing computational resources only where additional detail is required. This ensures particularly high recall of thin structures, such as rods and wires, which represent critical obstacles in underwater environments. Additionally, wavemap employs a “saturated region skipping” mechanism, efficiently skipping updates in areas where occupancy probabilities have already converged to the same value as the measurement update. This significantly reduces computational load in regions with partial or infrequent changes.

Wavemap’s hierarchical and always synchronized multi-resolution representation also provides significant performance benefits in downstream applications. In navigation tasks, it enables multi-resolution search-based planning, hierarchical traversability checking in sampling-based planners, hierarchical frontier extraction for exploration, and low-latency reactive collision avoidance. For inspection, wavemap’s wavelet-compressed maps can be efficiently transmitted over bandwidth-constrained and unreliable communication channels and naturally support progressive loading and interactive refinement over ROIs.

## 3 Proposed framework

**FIGURE 2:**
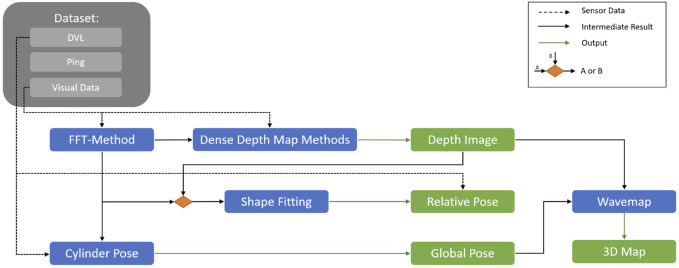
Overview of the proposed general framework for localization and mapping for UUVs operating in dynamically changing environments.

This section presents the full framework and discusses each of the pipeline’s components in detail. In particular, it covers how the robot’s net-relative pose, depth images, and global pose are estimated and how they are combined to create a 3D map of the net pen’s inspected area (Figure 2).

### 3.1 Field trials and datasets

**FIGURE 3:**
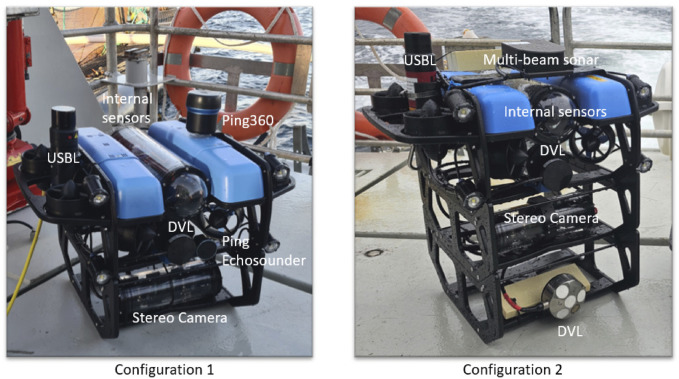
A multi-modal sensor platform for data collection and autonomous operations: BlueROV2 with integrated sensors used in the field trials.

Field trials have been performed in two different locations, namely Rataren (October 2023, Cage 2) and Singsholmen (August 2024, Cage 15), at SINTEF’s ACE facilities SINTEF (2023). The vehicle in Configuration 1 has been deployed during trials in 2023, while the system in Configuration 2 has been deployed for field trials in 2024 as shown in Figure 3 and described in Ohrem et al. (2025). The BlueROV2 with integrated sensors (Ping Echosounder, Ping360, Waterlinked DVL, Nortek DVL, Stereo Camera, Mono Camera, Multi-beam sonar), has been deployed and commanded to execute both manually controlled motions and net-relative autonomous navigation using Waterlinked DVL measurements (Haugaløkken et al., 2024). The specifications of the camera systems of the robotic systems are discussed in detail in (Ohrem et al., 2025), while four Lumen Subsea Lights were set on during the data acquisition (see Figure 3). As shown in Figure 3, Configuration 2 does not integrate the 360 Ping sonar while additional sensors such as Nortek DVL and Multi-beam sonar are integrated compared to Configuration 1. Note that in this study while we have considered datasets from both configurations of the system, we did not utilize DVL measurements from Nortek and the Multi-beam sonar.

During the trials, the net pen contained approximately 190,000 Atlantic salmon. Several datasets have been recorded in different locations inside the net pens. All datasets are logged with time-synchronization using ROS (Robot Operating System). The two field trials show contrasting biofouling levels, with the 2023 net pen being nearly biofouling-free and the 2024 net pen exhibiting moderate levels of biofouling. More information about the experimental setup and access to the datasets can be found in (Ohrem et al., 2025). The net’s grid cells during the trials were 20 mm × 20 mm and 27.5 mm × 27.5 mm in 2023 and 2024, respectively. In this paper, results are presented for several cases in which the vehicle was commanded to perform net-relative navigation at different depths and distances from the net. The following case studies are investigated; note that in this paper results are presented for Case 1 - Case 8, while cases from additional datasets have been presented in the Supplementary Appendix:

Case 1: Net-relative autonomous navigation at 3 m depth, with varying speed and reference distances set to 1 m, 2.1 m and 1.4 m (data from field trials in 2023),
Case 2: Net-relative autonomous navigation at 1.8 m depth, with varying speed and at a constant reference distance of 2 m (data from field trials in 2023),
Case 3: Net-relative autonomous navigation at 2.5 m depth with UUV speed of 0.1 m s^−1^, with reference distances set to 1 m and 1.5 m (data from field trials in 2024),
Case 4: Net-relative autonomous navigation at 4.8 m depth with UUV speed of 0.1 m s^−1^, with reference distances set to 1.5 m and 1 m (data from field trials in 2024),
Case 5: Net-relative autonomous navigation at 2.2 m depth with UUV speed of 0.2 m s^−1^, with reference distances set to 1 m and 1.5 m (data from field trials in 2024),
Case 6: Net-relative autonomous navigation at 2.4 m depth with UUV speed of 0.1 m s^−1^ with reference distances set to 1 m and 1.5 m (data from field trials in 2024),
Case 7: Manual navigation by an experienced operator at changing distances ranging from 0.5 m to 5 m (data from field trials in 2023), and
Case 8: Net-relative autonomous navigation at 5.2 m depth with a UUV speed of 0.2 m s^−1^, with reference distances set to 1 m and 1.4 m (data from field trials in 2024).

### 3.2 Net-relative pose estimation

The first component of the proposed framework aims to estimate the net-relative position of the UUVs inside the net pen. Note that (visual) SLAM methods require that unique features can reliably be matched across subsequent frames (odometry) and when revisiting the same place (loop closure). Fish nets and their support structures generate a very large number of features that correspond to different 3D points but have identical descriptors (environmental aliasing). Fish farms generally contain very few or no non-repetitive visual landmarks (Kelasidi and Svendsen, 2022). Furthermore, the fish themselves are visually striking but moving. This makes it very challenging to achieve sufficiently high recall and low false-positive rates to accurately estimate an ROV’s pose while robustly rejecting the dynamic features corresponding to fish–especially under real-time constraints. Furthermore as mentioned earlier, DVL sensors are sensitive to occlusions and erroneous measurements caused by moving fish (Amundsen et al., 2022). The accumulation of drift can be slowed down by relying on a high-end IMU, but this solution is very expensive and only extends the time frame within which the drift remains acceptable. This motivated our work to obtain relative pose estimates from camera images, to remove the dependency on DVL and accurate IMU measurements.

**FIGURE 4:**
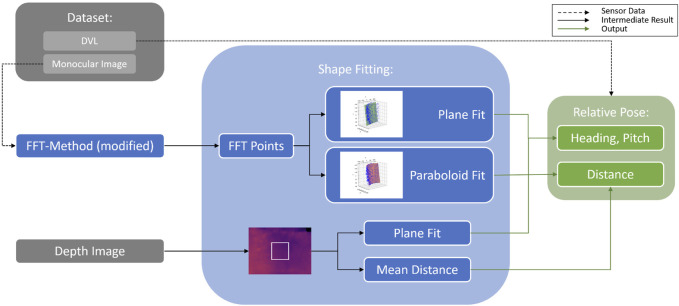
Relative pose estimation from either FFT generated points or from depth images.

**FIGURE 5:**
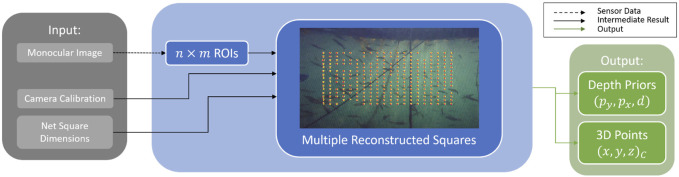
Modifications on the FFT-Method, resulting in depth priors and 3D point estimates.

As shown in Figure 4, the relative pose can be obtained either from acoustic sensors (e.g., DVL (Haugaløkken et al., 2024)) or by utilizing vision-based methods such as the FFT (i.e., FFT points), TRU-depth, and RMV (i.e., depth images). Besides using the estimated net-relative distances from the modified FFT-based method as priors for the depth estimation methods, as described in the following section, the obtained 3D points have also been used to estimate the robot’s relative pose. As shown in Figure 5, the FFT-based method proposed by Schellewald et al. (2021) has been modified in this paper to obtain multiple distance estimates to nets with a known net grid size. In particular, instead of outputting a single pose estimate, the modified version outputs multiple distance estimates, at known pixel locations (e.g., center of the ROIs spanning in the area of the image after disregarding 50 pixel around the image, as shown in Figure 5). Given the camera calibration, these can also be transferred to 3D point estimates. This is achieved by not only computing the FFT of a single ROI but also 20×15 ROIs, thus recreating multiple net squares and estimating the distance to each of them (see Figure 5). By fitting a plane to the 3D points, utilizing the least-squares method, one can compute the net-relative heading and pitch. The outcome of the modified FFT-based method provided the priors (distances) and then by applying plane and parabolic fitting and using the camera calibration parameters, it is possible to estimate the relative pose of the UUV (see Figure 4).

**FIGURE 6:**
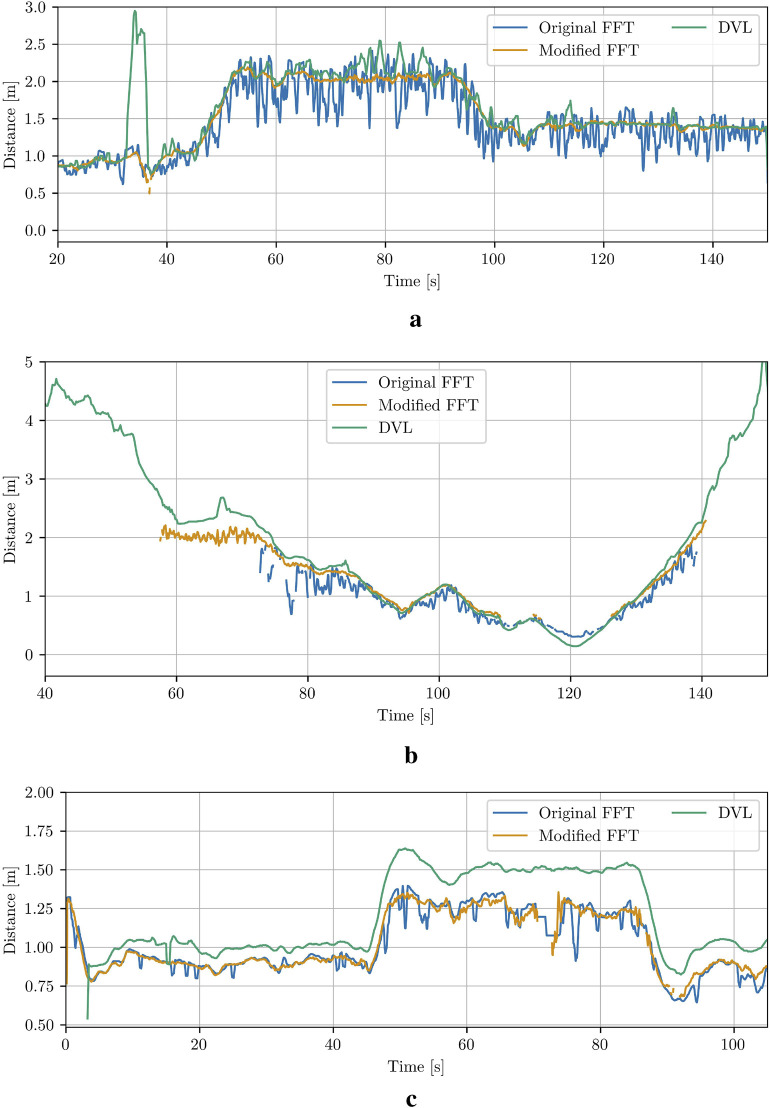
Comparison between the original and the modified FFT-based method for net-relative pose estimation in Case 1, Case 7 and Case 8. **(a)** Case 1. **(b)** Case 7. **(c)** Case 8.

As it can be seen in Figure 6, the resulting relative estimates are less noisy, and more precise than the results of the original method, therefore, in this paper, we will only compare the results from the modified version with the results from the other methods. From the comparison results shown in Figure 6c, it is obvious that both the original and the modified FFT-based methods underestimated the distances (especially when the robot keeps further distance from the net pen) compared to the results from 2023 (Figure 6a). The biofouling growth presence during the trials in 2024 could explain such observed errors since this will directly result to errors on the measured actual net grid size required from the FFT-based method. Other source of observed errors could be related to precise camera calibration, which can be investigated extensively in future studies.

To reduce runtime, the re-scaling of the ROI, as implemented in the original method (Schellewald et al., 2021), was omitted. This omission becomes evident when estimating distances close to the net ( d<70 cm), as the ROI becomes too small, resulting in the modified method failing to provide estimates (see Figure 6b). Note that as mentioned earlier the FFT-based method for pose estimation requires knowledge of the net grid size and this consequently affects the choice of ROI. Therefore, for the results obtained in this paper, ROI of 300×300 pixels and 512×512 pixels has been adapted for the data with net grid size of 20 mm × 20 mm (data from 2023) and 27.5 mm × 27.5 mm (data from 2024), respectively. The ROI size could be further optimized to maximize the detectable range or dynamically adjusted using DVL or Ping measurements, thereby eliminating the need for manual or iterative ROI size optimization. Interestingly, despite the lack of ROI resizing, the modified method performs better at the range limit of the original method, as shown in Figure 6.

### 3.3 Depth image prediction

In fish farming environments, estimating the net location is essential for understanding the surrounding environment for both self-localization and inspection purposes, meaning that, in addition to focusing on net-relative poses, there is also interest in obtaining dense depth information, particularly for scene reconstruction and mapping purposes. Several methods have been demonstrated to be effective in other underwater environments (Ebner et al., 2024; Skaldebø et al., 2024). This paper will discuss in detail the integration of two methods, TRU-depth (Ebner et al., 2024) and RMV (Job et al., 2024) respectively, into the underwater localization and mapping framework. The TRU-depth method trains on real data from the underwater domain, while the RMV method fine-tunes on synthetic data from aerial photogrammetry. We compare and adapt the two methods due to the challenging nature of the underwater domain in combination with semi-transparent net pens. On a high-level, both methods employ an encoder-decoder architecture, and the embeddings of the encoder are fed into a vision transformer to then produce metric depth predictions in the decoder.

Since the TRU-depth method is tailored to the underwater domain, it presents a natural choice for our problem as well. However, applying the method to images that primarily contain a net is challenging. As mentioned earlier, the method requires RGB images and additional point priors as inputs, necessitating a robust approach for generating these sparse depth estimates. Meaning that the TRU-depth network, not trained for such particular applications where we have structures with repeated textures requires a relatively large and consistent number of depth priors (the minimum number of required priors are set to 10). Classic feature-matching methods often fail when applied to repetitive, regular structures such as net grids (see Botta (2024) where both classical feature-matching and dense-matching approaches have been adapted and tested on the datasets obtained from industrial scale fish farms). The modified FFT-based method has therefore been used instead. This method provides reliable, uniformly distributed, and accurate net-relative distance estimates, which are then used as priors for the TRU-depth network (see Figure 2). Note that the TRU-depth method rescales the images to 320×240 pixels; therefore, the FFT-based priors were scaled accordingly to match the expected pixel locations for the priors. With these priors, the TRU-depth method was able to generate the dense depth images that accurately represent the 3D shape of the net pen. In an attempt to further enhance the results, the network was re-trained on images from the newly obtained dataset. Note that due to the lack of an absolute ground truth, DVL net-relative distance measurements were used to create uni-colored depth maps as ground truth for the re-training.

Since the FFT-based method can only detect the net, the TRU-depth network solely focused on the net as well, which disregards fish or other objects that might partially occlude the view. This demands research to explore how alternative methods would respond when a larger structure occludes a significant portion of the net, preventing the FFT-based method from detecting it in those areas. In particular, in cases where the net is not in the field of view (i.e. when approaching or leaving the net), fish pass the field of view, or biofouling occludes the net-grid structure, an alternative solution is required.

To address such situations, the RMV method is adapted (Job et al., 2024). As described previously, the method is designed to operate with few depth priors and can therefore provide dense depth predictions in situations where the FFT-based method fails completely or partly to provide sufficient number of priors required from TRU-depth method. In case that the FFT-based method fails completely, very sparse depth sources such as DVLs can be used as a replacement.

### 3.4 Global pose estimation

For the case studies for which the modified FFT-based method provided robust relative pose estimates (e.g., no occlusions from fish), the data has also been utilized to estimate the global pose of the UUV. Note that direct measurements of the global pose of the UUV from relevant sensors can also be used, provided the measurements are precise and readily available during UUV operations.

**FIGURE 7:**
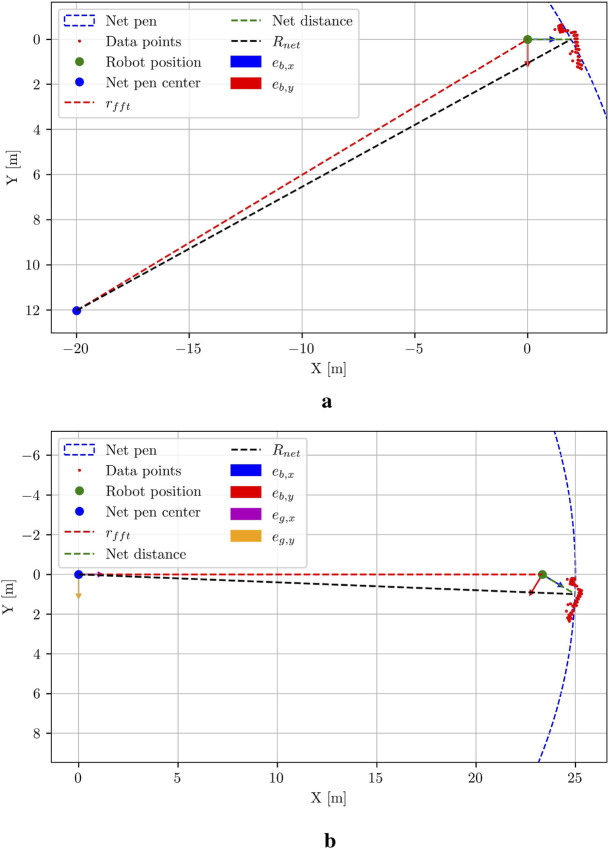
Estimation of the global radial coordinate. **(a)** The resulting net pen radial coordinate $r_{\mathrm{fft}}$ using the known net pen radius $R_{\mathrm{net}}$ in xy -body coordinates (coordinate frame unit vectors shown as $e_{b}$ ). **(b)** The resulting global radial coordinate $r_{\mathrm{fft}}$ in the global coordinate frame shown with unit vectors $e_{g}$ , which is based on the radial estimates obtained in $e_{b}$ . Note that $\rho^{k}$ is set to zero here, as this coordinate is obtained from integration of DVL velocities.

The global pose is defined as the robot’s 3D position and orientation within a fixed reference system in the net pen. The chosen coordinate system is aligned with the centerline of the net pen and is positioned at the water level, with the z -axis oriented downward and the angular coordinate ρ of the global coordinate system is defined such that $\rho^{0}=0$ corresponds to the robot’s starting location at time step k=0 . The points obtained from the modified FFT-based method are fitted to a cylinder of known diameter (e.g., the cage diameter provided from the fish farm) of 50 m (Ohrem et al., 2025) under the assumption that the net pen exhibits no deformation. Note that this is a reasonable assumption, given that the deformation is generally small and the results demonstrate the efficacy of the simple method for global pose estimation proposed in this paper. In this work, at least 30 detection points are required. This threshold was found to yield reliable estimates. The radial coordinate $r^{k}$ of the global coordinate system at time step k is estimated by fitting a circle to the obtained points projected onto the xy -plane in body coordinates. This is visualized in Figure 7a. Subsequently, the global position vector in Cartesian and polar coordinate system is $p_{g}^{k}=\left(x^{k},y^{k},z^{k}\right)=\left(r^{k},\rho^{k},z^{k}\right)$ , where the superscript k denotes the time step and the subscript g the global frame. The relative heading $\psi_{\mathrm{rel}}^{k}$ at the time step k is computed by transforming to the global frame. Since the robot’s roll and pitch were controlled to be zero during the trials, the problem reduces to 2D, simplifying the estimation. The global attitude then simplifies to $\eta^{k}=\left(0,0,\psi^{k}\right)$ . Note that this is a common process for UUVs with integrated roll and pitch stabilization modes.

The angular coordinate $\rho^{k}$ cannot be obtained directly from the FFT output and is therefore initialized to $\rho^{k}=0$ for the first computation step, as illustrated in Figure 7b. The angular coordinate of the UUV is obtained by utilizing the DVL velocities in body frame $v_{b,DVL}^{k}$ , since these measurements were much less noisy than the IMU sensor data of the BlueROV2. In particular, integrating the DVL velocities over one time step results in a new angular coordinate estimate $\rho^{k+1}$ . The $z^{k}$ -coordinate is obtained directly from the pressure sensor. The rotation matrix $R_{g,b}^{k}$ rotates the horizontal DVL body velocity to the global frame, which is then used to obtain a new position estimate $p_{g}^{k+1}$ through integration:

$$R_{g,b}^{k}=\left(\begin{array}{ccccc} \cos\rho^{k} & & -\sin\rho^{k} & & 0\\ \sin\rho^{k} & & \cos\rho^{k} & & 0\\ 0 & & 0 & & 0 \end{array}\right)$$ (2)

$$p_{g}^{k+1}=p_{g}^{k}+\Delta t\cdot R_{g,b}^{k}v_{b,DVL}^{k}$$ (3)

$$\rho^{k+1}=\mathrm{atan2}\left(y_{g}^{k+1},x_{g}^{k+1}\right)$$ (4)

**FIGURE 8:**
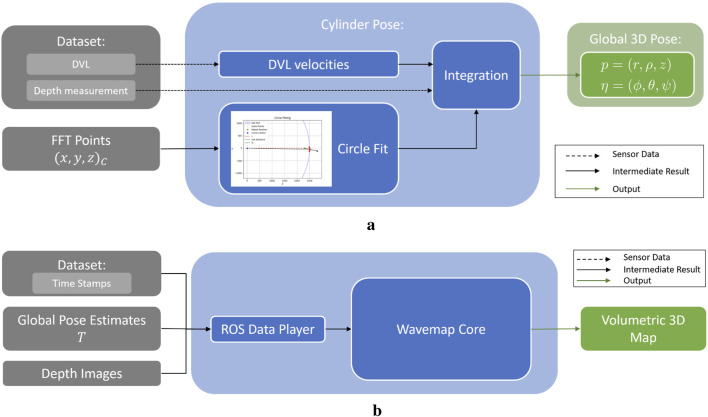
Global pose and mapping pipeline visualisation. **(a)** Integration pipeline to obtain a global pose estimate of the UUV within the net pen. **(b)** Pipeline to obtain volumetric 3D maps utilizing the wavemap method.

The radial coordinate $r^{k+1}$ is obtained with the computed $r_{\mathrm{fft}}^{k+1}$ from FFT for the current time step k , effectively rotating the situation from Figure 7b according to the velocity integration. The global heading angle is calculated as $\psi^{k}=\rho^{k}+\psi_{\mathrm{rel}}^{k}$ . Combining these results yields the global position $p^{k}$ and orientation $\eta^{k}$ estimates for the current time step k (see Figure 8a).

### 3.5 3D map representation

In this paper, two mapping approaches were tested: first, stacking of RGB point clouds generated from global pose estimates and camera images, which allowed for visual inspection of stacking quality and, by extension, the precision of the pose estimates. Second, the wavemap method was applied (see Reijgwart et al., 2023) to evaluate whether this technique, in combination with the predicted depth images, can provide a valuable mapping solution for underwater applications. All methods have demonstrated real-time capability (Ebner et al., 2024; Reijgwart et al., 2023), suggesting they offer promising directions for future development.

Figure 8b presents the pipeline to obtain volumetric 3D maps utilizing the wavemap method. To integrate the 3D pose estimates, each estimate, initially represented as {t=(r,ρ,z),R=(ϕ,θ,ψ)} , was transformed into a 4×4 homogeneous transformation matrix T . Additionally, the timestamps of the different pose estimates were extracted from the dataset. Utilizing a ROS data player, the wavemap method was then executed using each T and its corresponding depth image, generated by dense depth map estimation methods using FFT priors, along with the associated timestamps (see Figure 8b). This resulted in an incrementally built 3D volumetric map.

## 4 Results

This section presents evaluations across four key areas: relative poses, depth image predictions, global pose estimates, and mapping. To improve clarity and reduce noise, a sliding window smoothing filter has been applied to the plots of relative distance and orientation measurements. This technique enhances trend visibility and data variation by averaging points within a defined sliding window, offering a better representation of the methods’ performance. In contrast, the results for depth images, global pose estimates, and mapping are presented without smoothing to maintain the integrity of the raw data.

### 4.1 Net-relative pose estimation

The distance measurements from the DVL and the forward-facing ping echo sounder are compared with distance estimates obtained from the modified FFT-based method, the TRU-depth and the RMV. Note that to qualitatively compare the results from depth imaging methods, a method for extracting relative pose from depth images is proposed. In particular, the mean distance from the image center (choosing 100×100 pixels center region in Figure 4) is used as a distance estimate. For angular estimation, a least-squares planar fit is employed (this sub-pipeline is also illustrated in Figure 4). While this approximation is adequate for the data of the runs considered in this study and serves the purpose of comparing the obtained results, it is not a robust solution for determining net-relative pose of an UUV from depth images in general.

**FIGURE 9:**
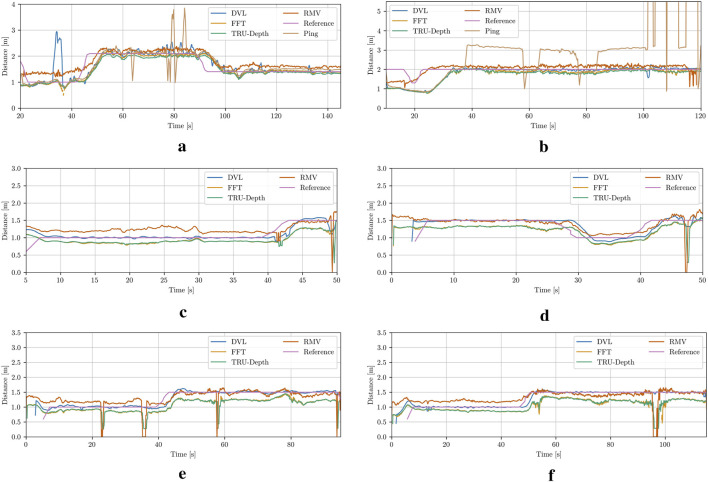
Net-relative distance comparison results using the estimation from the DVL plane approximation, modified FFT, TRU-depth utilizing all the obtained FFT priors, RMV utilizing up to 5 FFT priors, distance from the ping sensor and the reference, which corresponds to the desired input to UUV to follow constant distance from the net pen. **(a)** Case 1. **(b)** Case 2. **(c)** Case 3. **(d)** Case 4. **(e)** Case 5. **(f)** Case 6.

The results for the different case studies can be seen in Figure 9 and in the Supplementary Appendix. Since the DVL measurements are used as a reference signal by the UUV’s controller, the vision-based methods are compared to these values due to the lack of additional ground truth data. The results generally show a close alignment among the methods, with particularly strong agreement between the TRU-depth and FFT results for the majority of the investigated cases. It is quite evident that the TRU-depth method slightly underestimates the distance when compared to DVL measured signal, while RMV overestimates the obtained net-relative distance. The maximum error observed from results of RMV is of approximately 0.5 m which is in the range of most of the acoustic sensors utilized in underwater domain (Kelasidi and Svendsen, 2022). However, we can see that when the FFT method is not able to provide the required number of priors for the TRU-depth method (e.g., net occluded from obstacle in front of the camera), then this method is not able to estimate the net-relative distance, while RMV which required few priors provides smooth distance estimates (see Case 5 and Case 6 in Figure 9). As expected due to the presence of fish, it is notable that both acoustic sensors exhibit clear measurement outliers. This indicates the advantage of utilizing vision-based methods for robust localization in challenging underwater environments such the ones faced in fish farms.

**FIGURE 10:**
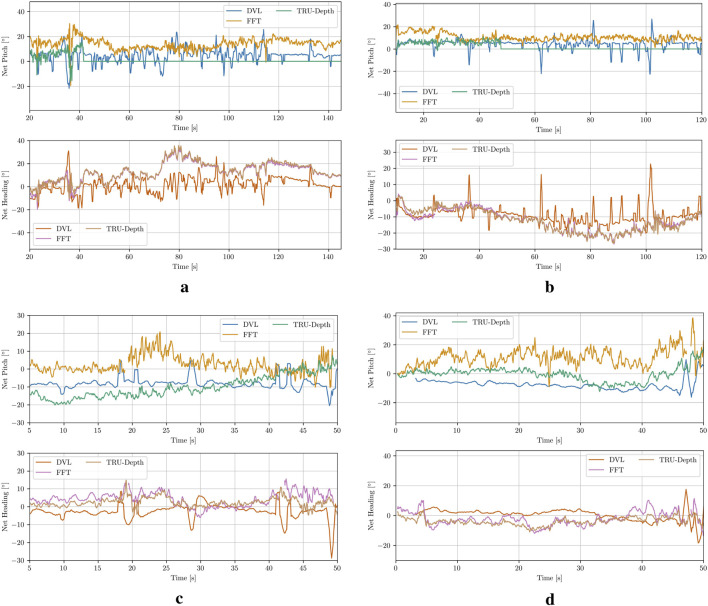
Net-relative orientation results using the estimation from the DVL plane approximation, modified FFT and TRU-depth utilizing all the obtained FFT priors. **(a)** Case 1. **(b)** Case 2. **(c)** Case 3. **(d)** Case 4.

In Figure 10, the comparison between the measured relative orientation computed from the DVL beams (Amundsen et al., 2022) and the relative orientation estimates calculated from the FFT points, as well as the TRU-depth-generated depth images, is presented. As shown, the overall trends are consistent, although the differences in relative orientation are larger than those observed in the relative distance estimates discussed above. It is also evident that all methods for obtaining relative orientations exhibit a significant amount of noise, with the acoustic sensor showing the most. Note that due to the lack of accurate ground truth data, a definitive assessment of which method provides a more precise estimation of the net’s relative orientation is not possible. Generally, the increased noise or variability in the results could partially be attributed to limited tuning of the heading controller during the trials. Future improvements could include better tuning of the controller to enhance performance.

Overall, the net-relative pose estimation results presented in this paper highlight the error-proneness of acoustic sensors in fish farming environments, as well as the capability of vision-based systems when operating close to or interacting with net structures. This underscores the importance of investigating vision-based methods for operations in fish farms, which often require net-relative control strategies.

### 4.2 Depth images

**FIGURE 11:**
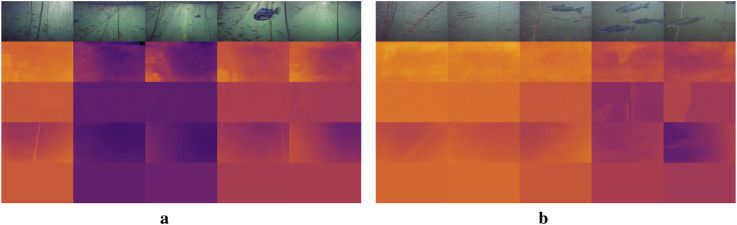
Comparison of depth images. Brighter colors correspond to smaller depth values, darker colors to larger values. The color range represents a depth range of 0 m–4 m for all depth predictions. 1st row: RGB image; 2nd row: TRU-depth; 3rd row: Retrained TRU-depth; 4th row: RMV method 5th row: DVL. **(a)** Case 1. **(b)** Case 3.

This section compares the depth imaging results of TRU-depth, the retrained TRU-depth and RMV models. From the resulting depth images in Figure 11, it is clear that the TRU-depth network does not detect fish in front of the net, which is to be expected since the network is only given net-based FFT priors (see Section 4.2). Note that the retrained TRU-depth network, trained using the flat DVL depth images as ground truth, shows a pronounced flattening effect after just one training epoch. This flattening is expected due to the use of single-value depth images as supervision signal. The retraining does not significantly affect the overall distance estimates to the net but essentially removes the capability to estimate the net’s relative orientation. Overall, the obtained results showcase the TRU-depth network’s effectiveness when provided with accurate priors, even in environments with few distinctive features and transparent structures not seen in its training data. This underscores the method’s significance for fish farming operations, highlighting its potential to perform reliably in such challenging conditions.

**FIGURE 12:**
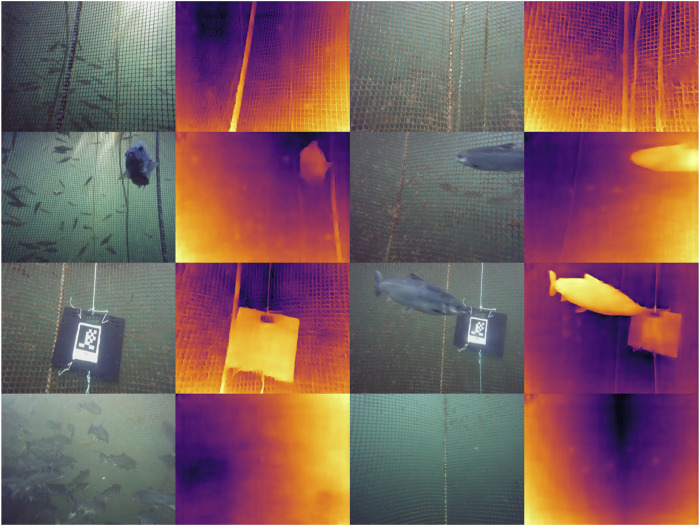
Alternating RGB input images and metric depth prediction. The depth predictions are consistent: The ropes, fish, and AprilTags are clearly visible in front of the net grid pattern.

The RMV method shows qualitatively better performance in identifying the net, ropes, fish in front of the net and other objects attached to net (i.e., AprilTags). This is shown in Figures 11, 12, where fish and structures in front of the net are often visible. If close enough, even the grid structure of the net is visible, which is essential for inspection of the net integrity. Overall, the results highlight the capability of this method to generalize to the underwater domain. We attribute the strong relative scene depth generalization capabilities to the pre-trained DepthAnythingV2 (Yang et al., 2024b) weights in combination with the metric predictions from the FFT priors coming from the fine-tuning as described by Job et al. (2024).

**FIGURE 13:**
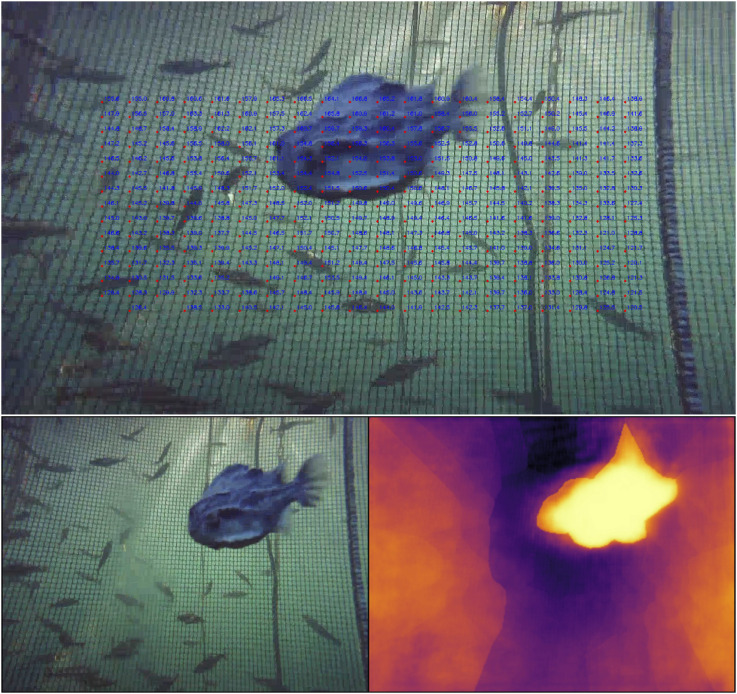
The FFT method based priors under partially occluded conditions. The net-distances are labelled within the image in centimetres (top plot) and the TRU-depth depth images generated by utilizing manually generated FFT-based priors (bottom row).

The main limitation of integrating FFT-based priors into the depth prediction methods is that it is only possible to obtain priors from the net pen. In the future, fusing in additional priors from classical stereo-matching or other methods to improve the detection of fish and other structures/objects could enable more comprehensive depth predictions. A proof of concept has been demonstrated for TRU-depth by manually creating priors for both fish and the net (see Section 4.2) and applying the TRU-depth network with these priors, as shown in Figure 13. Note that since the RMV method requires a small number of priors, we deem it also feasible to fuse very few priors from, e.g. DVL only and obtain metric depth predictions reliably.

### 4.3 Global pose estimation

**FIGURE 14:**
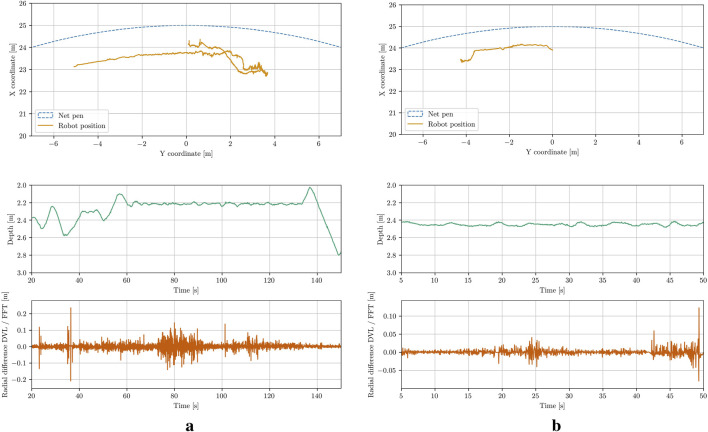
The trajectory estimation results for the UUV. **(a)** Case 1. **(b)** Case 3.

Assuming no deformations in the net pen and no pitch and roll of the UUV, the global position of the vehicle has been reconstructed using the DVL velocity measurements. The resulting trajectories are displayed in Figure 14. The top plot shows the trajectory from a top-down view, while the second plot illustrates the third dimension, depth. The third plot displays the difference between the radial coordinate obtained from integration, and the one derived from circle fitting. It is evident that, aside from brief periods, the errors between the integration estimate and the actual optical distance estimate are small. The few peaks in the error correspond to instances where the ROV changes direction rapidly due to control input adjustments, resulting in imprecise DVL measurements and blurred images that lead to less accurate FFT-based estimates.

**FIGURE 15:**
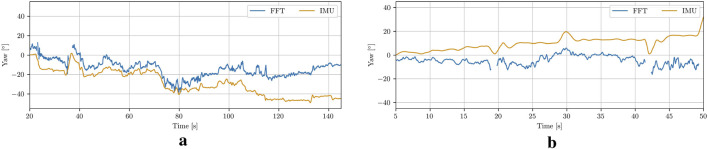
Global Heading comparison of the IMU measurements and the cylinder pose estimations. **(a)** Case 1. **(b)** Case 3.

To further evaluate the estimation accuracy, the calculated global yaw estimates have been compared with the onboard IMU measurements, as illustrated in Figure 15. Since the robot’s initial heading was arbitrary, the yaw-estimate plots are all shifted to start at zero. The observed drift between the estimated and measured values over time could be attributed to several factors, including imprecise estimates, integration errors, or sensor drift in the DVL. Sensor drift in the IMU itself also contributes to the discrepancy. Overall, the vehicle’s trajectory has been estimated in a manner that appeared consistent with the video data. Further evaluation through point cloud stacking indicated that the estimates are relatively accurate.

### 4.4 3D map representation

**FIGURE 16:**
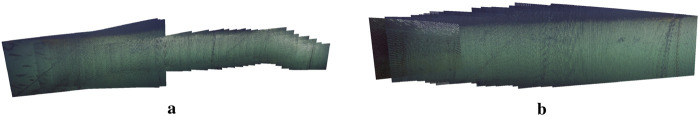
Stacked point clouds from image projection onto cylinder fittings. The vertical and diagonal ropes in the image visibly coincide, which shows the accuracy of the pose estimation. **(a)** Case 1. **(b)** Case 2.

To further assess the precision of the global pose estimations, the RGB data was projected onto the estimated net cylinder, and the resulting RGB point clouds were stacked, as shown in Figure 16. By observing different lines visible across multiple images, one can gauge the accuracy of the position estimates. A clear example is the diagonal rope visible on the left side of the stacked point cloud, which runs through multiple images and connects smoothly, even though the images were taken from different distances and orientations.

**FIGURE 17:**
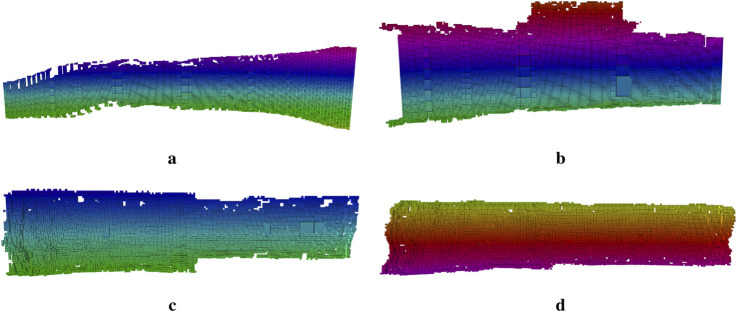
Volumetric maps generated by the wavemap method using the depth images generated by TRU-depth and the estimated 3D poses. Only the occupied voxels are shown, colored by their z-coordinate in the relative frame of the map.

**FIGURE 18:**
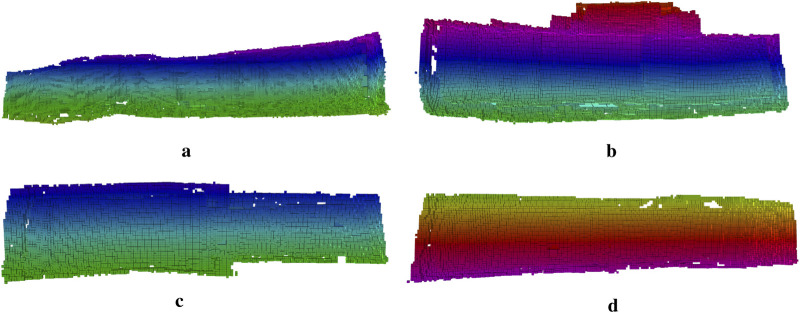
Volumetric maps generated by the wavemap method using the depth images generated by RMV and the estimated 3D poses. Only the occupied voxels are shown, colored by their z-coordinate in the relative frame of the map. **(a)** Case 1. **(b)** Case 2. **(c)** Case 3. **(d)** Case 4.

To assess the potential of using learning-based depth images for mapping and to evaluate the feasibility of applying the wavemap method (Reijgwart et al., 2023) in underwater environments, wavemap was used to fuse the depth images predicted by TRU-depth and RMV at their estimated 3D poses. We have adapted wavemap in this paper since (Reijgwart et al., 2023) presented an in-depth performance comparison between wavemap, octomap, voxblox and supereight2, where it is shown that wavemap achieves the highest recall and memory efficiency, while matching the state of the art in terms of overall accuracy and computational efficiency. The experiments in wavemap’s original paper include also comparisons integrating a depth image at various resolutions, which matches the technical setup in our current paper. The resulting 3D volumetric maps are shown in Figures 17, 18. Note that only the occupied voxels are shown, colored by their z -coordinate in the map’s coordinate frame. As can be seen, the reconstructed surfaces are smooth–indicating that wavemap effectively filters out the noise in the predicted depth images–and match the net’s expected curvature. When comparing Figures 17, 18, the TRU-depth- and RMV method -based 3D maps, respectively, we can observe few differences in the output representations. As previously mentioned, a distinct flattening effect is present in the depth predictions of the TRU-depth method, which would lead to expecting an increased consistency when constructing the 3D map of the net structure as the observations match more closely with the FFT prior points. However the resulting 3D maps using the depth predictions of the TRU-depth (see Figure 17) and the results using the RMV method (see Figure 18) are providing same level of information, making both methods very suited for 3D mapping of net-pens.

This paper shows that by fusing net-relative pose measurements with IMU and depth readings, our framework effectively eliminates most drift sources. However, the ROV’s absolute yaw angle remains unobservable and this error might therefore slowly grow over time. Previous work has shown that the accumulation of drift, and corresponding mapping errors, can effectively be avoided by performing pose graph optimization over a graph of volumetric submaps (Reijgwart et al., 2020). Extending our work to create and align a collection of wavemap submaps, in the same way voxgraph (Reijgwart et al., 2020) used a collection of voxblox submaps (Oleynikova et al., 2017), would allow drift along yaw to be eliminated as long as there is at least one irregularity or object that makes the map non-symmetric along the net pen’s cylindrical axis. This would be an interesting direction for future work.

In general, the results demonstrate the potential of using wavemap to fuse the predicted depth images into an accurate volumetric map. Since the depth prediction methods and wavemap both run in real-time (Ebner et al., 2024; Reijgwart et al., 2023), this map provides a live 3D reconstruction that can be used for increased situational awareness of an operator and facilitate autonomous navigation including global path planning (Reijgwart, 2024) and reactive collision avoidance Reijgwart et al. (2024). Note that wavemap was primarily designed for accurate, low-latency navigation, including reactive collision avoidance (Reijgwart et al., 2024). The reactive collision avoidance policy can also readily be used to enhance safety during manual piloting (Reijgwart et al., 2024). Given its geometric accuracy, wavemap maps are already well suited for structural analysis tasks. Extending the framework to include additional channels, such as reflectivity, color information or semantics is straightforward. This future work would allow it to be used to monitor non-geometric properties, including biofouling, as well.

### 4.5 Net-relative distance results

In general, obtaining absolute ground truth measurements in underwater environments is challenging. To address this challenge and obtain ground truth distances to the net, passive visual markers (i.e., AprilTags) have been placed directly on the net surface during the field trials in 2024. Note that since the visual markers are not visible in every frame thus continuous ground truth results are not feasible during the full autonomous net-relative navigation mission. In addition, occlusions from AprilTags will influence the results of all camera-based methods. However, having AprilTags attached on the net and using the camera intrinsic parameters, the physical size of the tags and the knowledge that the tag is printed on a rigid, flat plate, the relative pose of the camera to the tag can be estimated (Wang and Olson, 2016). In particular, adapting the methods proposed by Wang and Olson (2016), we identify the corners of the markers and extract the average depth values as ground truth in the rectangle described by the four points.

With the knowledge of the pose of visible AprilTags and extracting their depth values, it was possible to evaluate the depth prediction performance of all net-relative distance estimation methods investigated in this paper. The mean absolute error (MAE) metric is adapted for this comparison and it is given by

$$e_{\mathrm{MAE}}=\text{mean}\left(\left|d_{i}-d_{\mathrm{AprilTag}}\right|\right),$$ (5)

where $d_{\mathrm{AprilTag}}$ is depth of the AprilTag marker and $d_{i}$ is the depth of either the TRU-depth, the RMV, the DVL, the modified-FFT method or sensor in the image location of the AprilTag. Meaning that if the respective net-relative distance method performs adequately, the depth of the AprilTag should correspond to the predicted depth in the location of the marker and result in a low absolute relative error.

**FIGURE 19:**
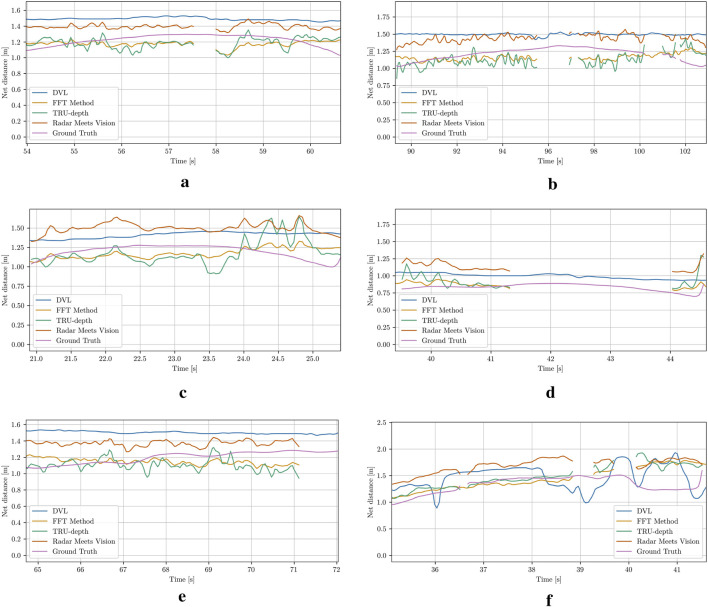
Ground truth distance from AprilTags mounted directly in front of the net, compared to DVL, modified FFT, TRU-depth and RMV method. All methods besides DVL are only plotted when the AprilTag is in view and its pose can be computed. **(a)** Case 5: A single marker is visible between 54 s and 61 s. The FFT-priors get lost between 57.5 s and 58 s. **(b)** Case 6: A single marker is visible between 90 s and 104 s. At 97 s a fish passes the field of view of the camera. **(c)** Case 9: A marker is visible between 21 s and 25 s. **(d)** Case 10: A single marker is visible between 40 s and 45 s. The FFT-method fails to provide priors for a prolonged time from 41.5 s. **(e)** Case 11: A single marker is visible between 65 s and 72 s. The FFT-method fails after 71 s, due to the tag influencing the readings. **(f)** Case 12: A single marker is visible between 35 s and 42 s. Fish are passing the camera view, which explains large changes in the net-distance predictions.

Figure 19 and Table 1 show comparison results of the distances estimated and performance in terms of the average absolute error from the DVL sensor measurements, modified FFT-based, TRU-depth and RMV methods, respectively. The errors reported in Table 1 refer to the average depth values within the AprilTag evaluation rectangle, while the number of frames with observable markers is shown in the last column of Table 1. The estimated depth distances are plotted for the time duration in which the AprilTags were visible in the recordings for several cases (see Figure 19). As the DVL returns a single point depth, it is assumed to have the same depth in the center, as well as the tag location. It is important to mention that no FFT-based priors can be generated directly on the AprilTag. However, since the net can be represented as a plane in each frame the net distance error introduced with the approximation for the FFT-based depth on the AprilTag is expected to be generally low. Therefore, since the modified FFT method returns only points on the net, the tag depth is estimated by the average of all FFT points closer than one tag size away. This heuristic was found to yield satisfactory results with markers of the 36h11-family and a tag size of 15 cm (Wang and Olson, 2016).

**TABLE 1:** MAE of the respective depth prediction method compared to the ground truth derived from the AprilTag marker.

MAE [m]	Modified FFT	TRU- depth	RMV	DVL	N. AprilTag frames
Case 5	**0.095**	0.132	0.173	0.269	158
Case 6	**0.089**	0.159	0.235	0.312	420
Case 9	**0.135**	0.207	0.281	0.205	127
Case 10	**0.074**	0.135	0.318	0.231	73
Case 11	**0.098**	0.147	0.187	0.320	157
Case 12	**0.189**	0.194	0.389	0.265	136
Avg. all cases	**0.113**	0.162	0.264	0.267	179

The results are averages over all frames, where the marker can be detected and each measurement method is available at the same timestamp. The best values are in bold, and the second-best values are underlined. The Radar Meets Vision method is abbreviated as RMV.

In Figures 19a,d,e dropout in depth estimates from the FFT method can also be seen, which is due to the April tag blocking the net-grid structure. From Figures 19b,f, it is also clear that fish can significantly influence the depth prediction output, which needs to be taken into account for applications in environments such as fish farms. An additional observation is that both the DVL sensor measurements and the RMV method almost always overestimate the net ground-truth distance. The net structure is not very dense; therefore, a hypothesis is that the DVL sensor systematically overestimates the distance. A similar hypothesis might be true for the RMV method: The depth priors are provided as circular blobs, which do not always precisely match the grid-like net-structure. The semi-transparent nature of the net might then lead to this systematic error.

In Table 1, we show the MAE of all methods in estimating the depth to the April Tag mounted on the net. On a high-level, the results show that the modified FFT-based method outperforms all other methods. The second-best method is the TRU-depth method. As discussed previously, the TRU-depth method follows the FFT-method very closely and does not incorporate so many visual features. The DVL and RMV methods both perform worse, which can mostly be explained due to the systematic error introduced by over-estimating the net-relative distance. Overall, the results show that errors calculated for all the investigated methods are smaller than 0.5 m (i.e., precision of a common acoustic sensors in underwater domain) with the average of all the investigated cases being approximately 0.2 m. The FFT-method consistently outperforms the other methods. The FFT-method and TRU-depth method also rely heavily on the presence and prior knowledge of the grid-like net structure, whereas the RMV and DVL methods can be used outside the fish-net context, and are therefore viable solutions in failure and transition phases around net pens of fish farms. Overall, these results emphasize the importance of multi-modal sensor modalities, especially in remote and challenging underwater environments.

### 4.6 Runtime

All runtimes are evaluated on the same computer, featuring an Intel 13700KF CPU paired with 64GB of RAM and an NVIDIA 4070Ti GPU. The most time-intensive part of the pipeline is the computation of the depth priors using the FFT method (Section 2.1). Fortunately, each patch can be processed independently, allowing us to parallelize this operation using a CPU thread pool. The TRU-depth and RMV depth predictions are computed on the GPU, while wavemap runs on the CPU with multi-threading. The relative and cylinder poses are each computed using a single thread on the CPU.

The average runtimes of our proposed pipeline’s components are shown in Table 2. The most important observation is that all components can run at 5 Hz or more, thereby being suitable for real-time operation on a UUV. The biggest bottleneck remains the FFT depth prior computation. Aside from optimizing its implementation, it could be worth investigating how the pipeline’s other components perform when provided with a smaller number of priors, since the FFT method’s runtime directly scales with the number of patches it evaluates. TRU-depth, for example, is very efficient but required very dense depth priors for applications in fish farms. In contrast, the network of RMV is more expensive but generalizes better and requires fewer priors to achieve high accuracy. Combining it with FFT method’s 300×300 px configuration already results in a capable real-time depth prediction pipeline, and it might still perform well with an even lower number of priors. As expected, computing the relative and cylinder poses is very fast. Finally, we see that wavemap is both efficient and updates the map with very low latency, making it well-suited to run in the background while supporting navigation and collision avoidance tasks.

**TABLE 2:** Comparison of method runtimes.

Method	Avg. Runtime (ms)	Number of ran frames
FFT (300x300px)	176.4	> 800
FFT (512x512px)	403.5	> 800
TRU-depth Network	1.13	> 3500
RMV Network	23.71	> 500
Relative Pose	0.083	> 10000
Cylinder Pose	1.251	> 10000
wavemap	31.8	50

## 5 Conclusion

This paper proposed a general, vision-based framework for underwater localization and mapping, and utilized a large dataset recorded in industrial scale fish farms. The framework leverages the FFT-based method to generate priors for the TRU-depth and RMV methods, which in turn provide the depth image predictions required for 3D mapping. Additionally, methods for obtaining net-relative and global pose estimates of UUVs have been proposed. The results demonstrate the potential of the framework to integrate the FFT-based method, depth image predictions and wavemap methods for applications in fish farming environments. It specifically showed that TRU-Depth and RMV methods can generate depth images from monocular images within these environments. Coupling the depth prediction methods with the wavemap method and 3D pose estimates, the pipeline enables the creation of detailed volumetric maps. The completeness and accuracy of these 3D maps highlight their potential for real-world applications in the underwater domain. While it would be valuable to directly compare our global pose estimation approach with state-of-the-art SLAM methods, we note that standard SLAM methods are fundamentally challenged in the highly repetitive and dynamic environment of fish farms. The lack of distinct visual features, frequent occlusions by moving fish and the flexible nature of net structures typically lead to frequent tracking loss and high drift, as reported in recent literature on the challenges in the underwater domain (Wang et al., 2023; Song et al., 2024). As a result, conventional SLAM methods are unlikely to provide meaningful baselines. Nevertheless, we acknowledge that a systematic evaluation of such methods, even if they perform poorly, could further illustrate the advantages and robustness of our proposed framework, and we leave this as an important direction for future work. In general, incorporating active loop-closure, global SLAM, or additional sensing modalities such as sonar or stereo vision could further increase the robustness of the proposed pipeline, particularly in low-visibility conditions. Alternative methods to obtain depth priors on fish or other distinct structures could also be combined with the FFT-based priors to enable depth image prediction methods and wavemap to comprehensively reconstruct 3D underwater scenes from monocular images. In the future, it would also be relevant to evaluate the performance of the fully integrated framework in datasets obtained in a controlled environment.

## Data Availability

Publicly available datasets were analyzed in this study. This data can be found here: https://arxiv.org/abs/2504.01790.
