# Supplementary material for: Leveraging learned monocular depth prediction for pose estimation and mapping on unmanned underwater vehicles
Source: Front Robot AI. 2025 Jun 26;12:1609765. doi: 10.3389/frobt.2025.1609765 (PMC12240768; doi:10.3389/frobt.2025.1609765)
Supplement: Supplementary file 1 [file Supplementaryfile1.pdf]

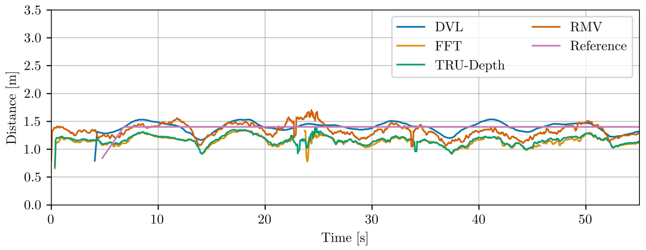

Figure 20a. Case 9

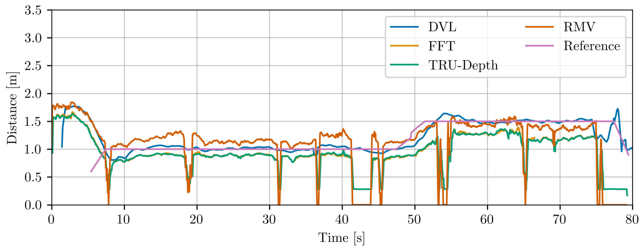

Figure 20b. Case 10

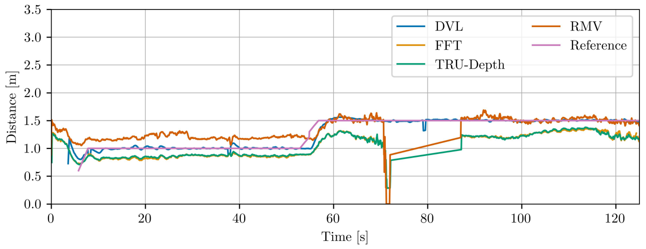

Figure 20c. Case 11

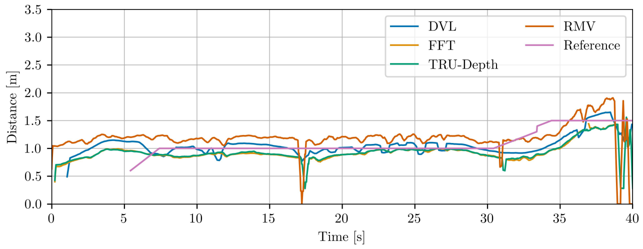

Figure 20d. Case 12

Figure 20. Net-relative distance comparison results using the estimation from the DVL plane approximation, modified FFT, TRU-depth utilizing all the obtained FFT priors, Radar Meets Vision (RMV) utilizing up to 5 FFT priors, distance from the ping sensor and the reference, which corresponds to the desired input to UUV to follow constant distance from the net pen.

APPENDIX

In this section, plots from additional datasets have been presented for the following cases:

Case 9:Net-relative autonomous navigation at $5.2\,\mathrm{m}$ depth with UUV speed of $0.3\,\mathrm{m\,s^{-1}}$, with reference distances set to $1.4\,\mathrm{m}$ (data from field trials in 2024)

Case 10:Net-relative autonomous navigation at $2.3\,\mathrm{m}$ depth with UUV speed of $0.2\,\mathrm{m\,s^{-1}}$, with reference distances set to $1\,\mathrm{m}$ and $1.5\,\mathrm{m}$ (data from field trials in 2024)

Case 11:Net-relative autonomous navigation at $2.3\,\mathrm{m}$ depth with UUV speed of $0.1\,\mathrm{m\,s^{-1}}$, with reference distances set to $1\,\mathrm{m}$ and $1.5\,\mathrm{m}$ (data from field trials in 2024)

Case 12:Net-relative autonomous navigation at $5.5\,\mathrm{m}$ depth with UUV speed of $0.2\,\mathrm{m\,s^{-1}}$, with reference distances set to $1\,\mathrm{m}$ and $1.5\,\mathrm{m}$ (data from field trials in 2024)

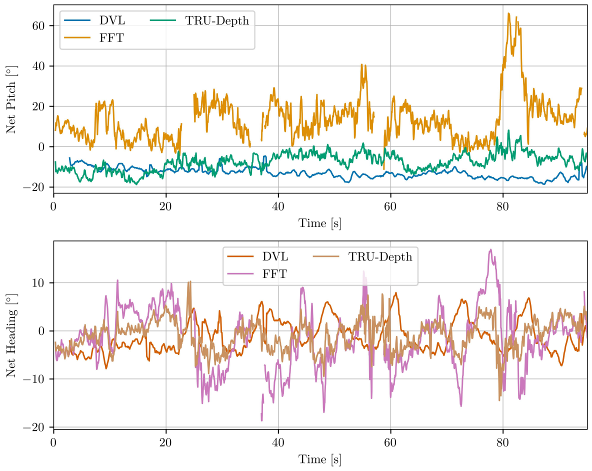

Figure 21a. Case 5

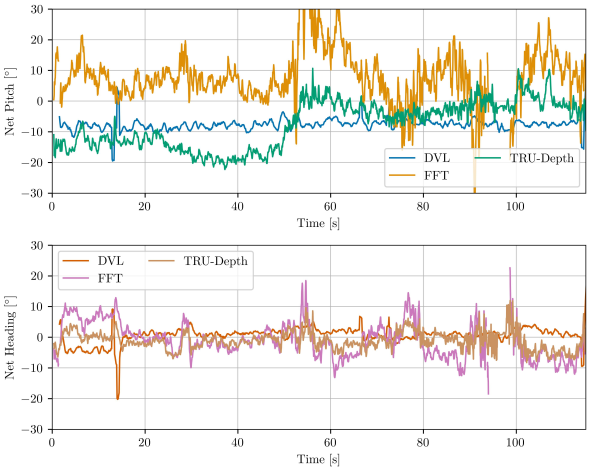

Figure 21b. Case 6

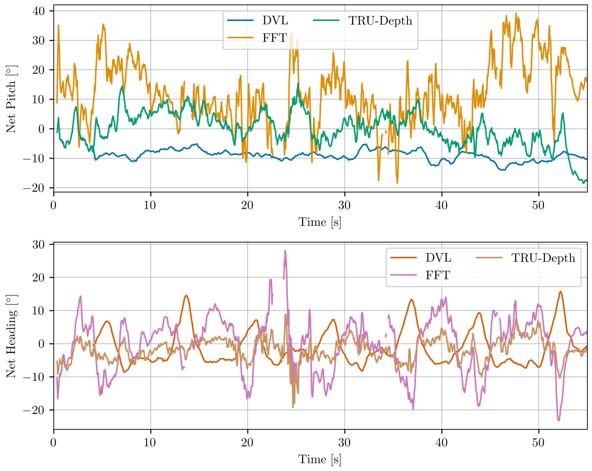

Figure 21c. Case 9

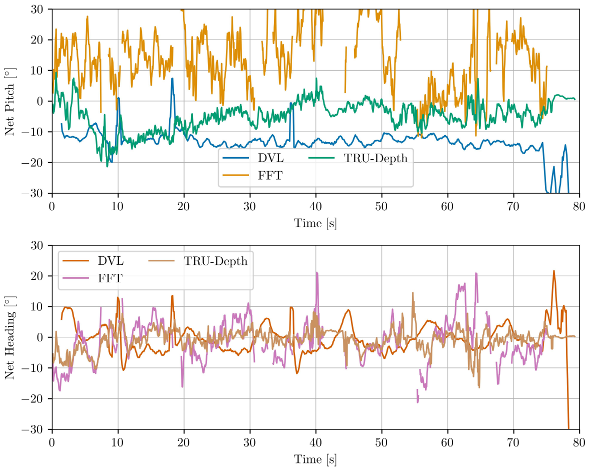

Figure 21d. Case 10

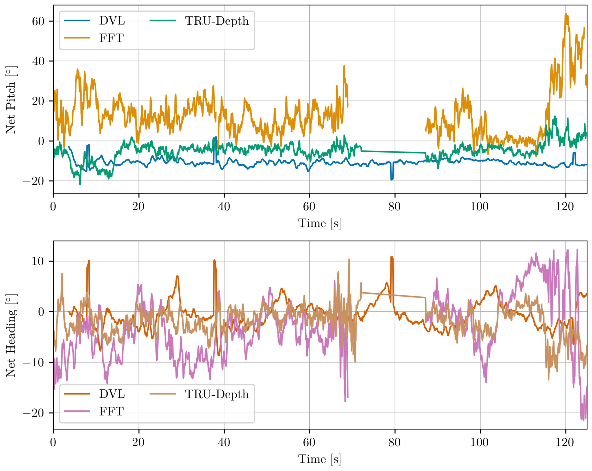

Figure 21e. Case 11

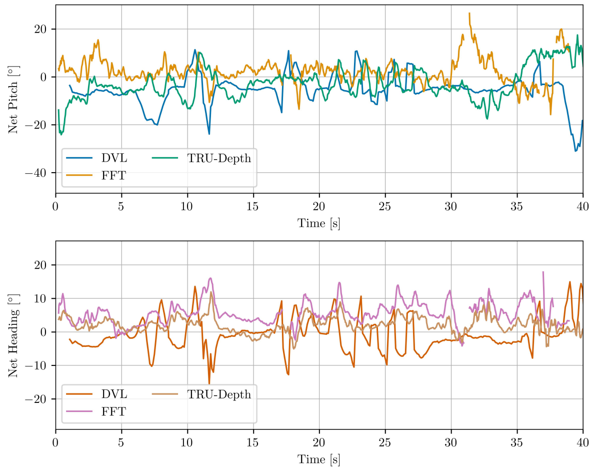

Figure 21f. Case 12

Figure 21. Net-relative orientation results using the estimation from the DVL plane approximation, modified FFT and TRU-depth utilizing all the obtained FFT priors.

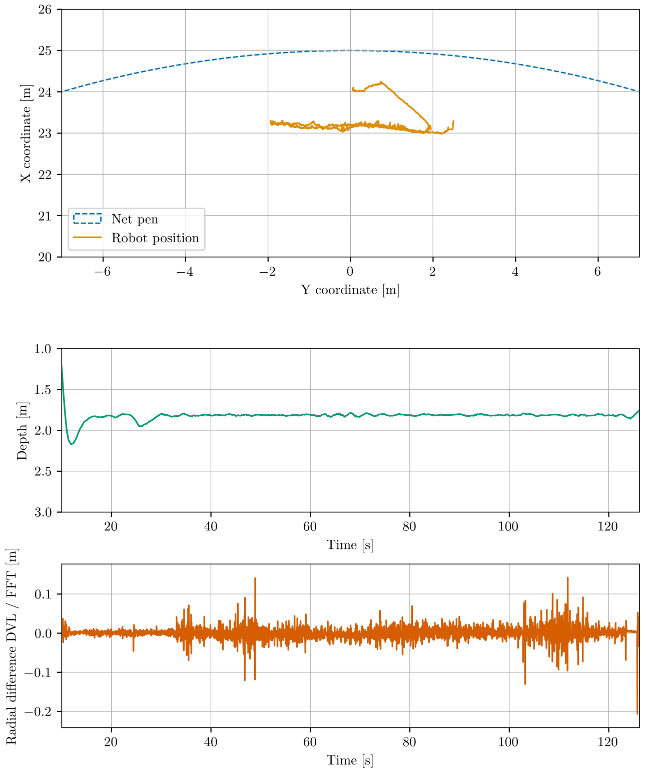

Figure 22a. Case 2

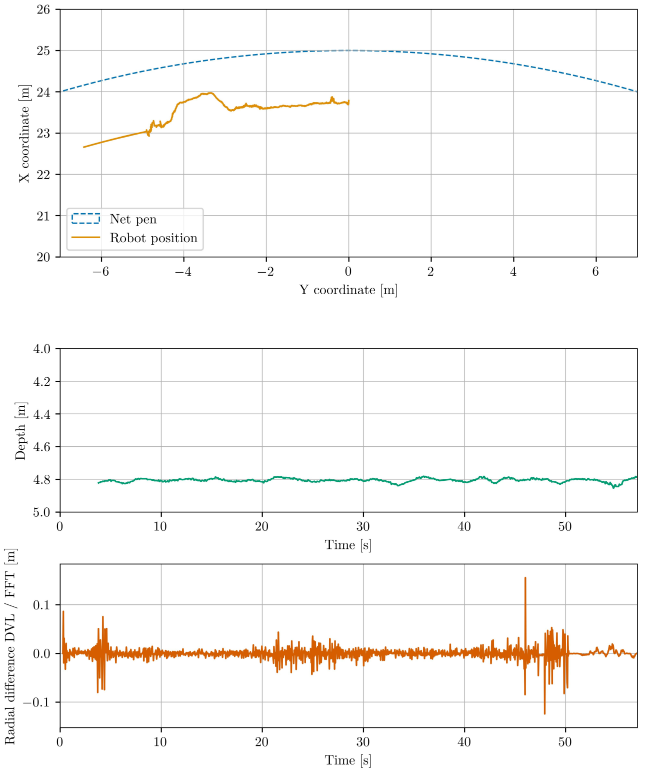

Figure 22b. Case 4

Figure 22. Trajectory estimation results for the UUV.

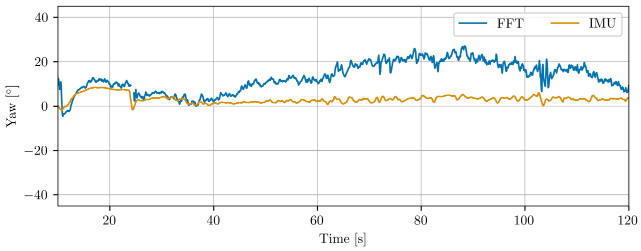

Figure 23a. Case 2

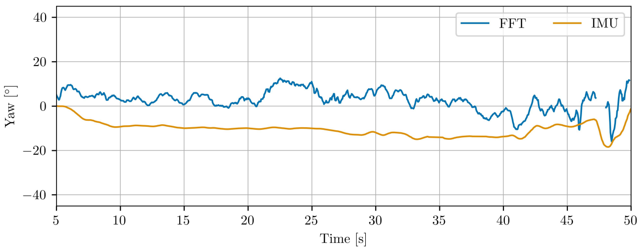

Figure 23b. Case 4

Figure 23. Global heading comparison of the IMU measurements and the cylinder pose estimations.
